# Geometrical Properties of the Nucleus and Chromosome Intermingling Are Possible Major Parameters of Chromosome Aberration Formation

**DOI:** 10.3390/ijms23158638

**Published:** 2022-08-03

**Authors:** Floriane Poignant, Ianik Plante, Zarana S. Patel, Janice L. Huff, Tony C. Slaba

**Affiliations:** 1 National Institute of Aerospace, Hampton, VA 23666, USA; 2 KBR, Houston, TX 77058, USA; ianik.plante-1@nasa.gov (I.P.); zarana.s.patel@gmail.com (Z.S.P.); 3 NASA Johnson Space Center, Houston, TX 77058, USA; 4 NASA Langley Research Center, Houston, VA 23681, USA; janice.l.huff@nasa.gov (J.L.H.); tony.c.slaba@nasa.gov (T.C.S.)

**Keywords:** Monte Carlo radiation track structure, high charge and energy (HZE) ions, ionizing radiation, chromosome aberration, nuclear architecture

## Abstract

Ionizing radiation causes chromosome aberrations, which are possible biomarkers to assess space radiation cancer risks. Using the Monte Carlo codes Relativistic Ion Tracks (RITRACKS) and Radiation-Induced Tracks, Chromosome Aberrations, Repair and Damage (RITCARD), we investigated how geometrical properties of the cell nucleus, irradiated with ion beams of linear energy transfer (LET) ranging from 0.22 keV/μm to 195 keV/μm, influence the yield of simple and complex exchanges. We focused on the effect of (1) nuclear volume by considering spherical nuclei of varying radii; (2) nuclear shape by considering ellipsoidal nuclei of varying thicknesses; (3) beam orientation; and (4) chromosome intermingling by constraining or not constraining chromosomes in non-overlapping domains. In general, small nuclear volumes yield a higher number of complex exchanges, as compared to larger nuclear volumes, and a higher number of simple exchanges for LET < 40 keV/μm. Nuclear flattening reduces complex exchanges for high-LET beams when irradiated along the flattened axis. The beam orientation also affects yields for ellipsoidal nuclei. Reducing chromosome intermingling decreases both simple and complex exchanges. Our results suggest that the beam orientation, the geometry of the cell nucleus, and the organization of the chromosomes within are important parameters for the formation of aberrations that must be considered to model and translate in vitro results to in vivo risks.

## 1. Introduction

Exposure to ionizing radiation is identified as one of the five main hazards of spaceflight [1]. Beyond low Earth orbit, astronauts are exposed to a constant flux of galactic cosmic rays and intermittent solar particle events. Galactic cosmic rays are composed of ions, notably protons, alpha particles, and high charge and energy (HZE) particles. When traversing biological tissues, these particles deposit energy along their path by interacting with atoms and molecules they encounter, mostly by ionizations and excitations. These events can directly induce deoxyribonucleic acid (DNA) single or double strand breaks (DSBs) or create chemical species such as the hydroxyl radical that can further damage the DNA. As cells attempt to repair the damage, fragments of DNA may be improperly repaired, leading to the formation of chromosome aberrations (CAs). If the cells survive the injury, they may divide, and transfer the CA onto their cell daughters, resulting in mutations that can develop into cancer over time [2,3,4]. Compared to terrestrial radiation (commonly γ-rays), HZE ions have a high linear energy transfer (LET), which characterizes the amount of energy deposited per unit path length. High-LET ions create clustered DNA breaks [5,6,7] exhibiting multiple closely located single and double DNA strand breaks, which are more difficult for the cell to repair, and thus favor formation of CAs. CAs are biomarkers of space radiation exposure [8,9]. Because chromosome instability is one of the primary drivers for tumor initiation [10,11,12,13], understanding and quantifying CA formation is of interest to estimate the risk of radiation-induced carcinogenesis in astronauts, or in other contexts such as biodosimetry in accidental irradiation exposure scenarios.

Over the past decades, many experiments have been performed in ground-based facilities to assess how simulated space radiation affects the formation of CAs and how they vary with physical or biological parameters. Experiments have consistently reported an increased relative biological effectiveness (RBE) for HZE ions compared to low-LET radiation (X-rays or γ-rays). These studies demonstrate an RBE peaking around an LET value of 100–200 keV/µm for various types of endpoints such as initial chromatin breaks [14,15], non-rejoined chromatin breaks [16], simple, complex, or total exchanges [17,18,19,20,21], and mutations [22,23,24]. These studies were conducted using a variety of normal cell lines (lymphocytes, epithelial cells, and fibroblasts) that have distinct nuclear sizes and shapes in vitro. Lymphocyte nuclei are relatively small and spherical, with a radius typically equal to 3 μm [25,26], and volumes between ~10 μm^3^ and 150 μm^3^. Epithelial cell nuclei have an ellipsoidal, elongated shape reaching an average volume of 220 μm^3^ to 240 μm^3^ [4,27]. Fibroblast nuclei are usually described with a similar shape as epithelial cells but with a larger nuclear volume usually ranging from 500 μm^3^ to 1000 μm^3^ [25,27,28,29,30], although a few studies reported smaller sizes depending on the cell line [31,32]. Volumes can also vary within the same cell population. For instance, the size of the nucleus for a dividing fibroblast varies with time, with a periodicity matching that of the cell cycle. The volume also varies for the same cell type taken from different individuals [30]. Additionally, the shape and volume of the nucleus vary depending on experimental conditions, and, in particular, whether cells are in suspension or attached to a substrate [33].

Several authors have suggested that the formation of CAs may be influenced by the size and shape of nuclei [4,32,33,34,35,36]. Chromosomes occupy fixed domains known as chromosome territories, whose size and shape are influenced by the nuclear geometry [25,37], with intermingling regions defined where chromosomes are more or less in close contact depending on the cell line [36,38,39]. DNA breaks are believed to move either by Brownian motion [40,41], or by active transport possibly towards repair centers [42], or by the effect of extrinsic forces mediated by the nuclear architecture [43]. After damage, chromatin undergoes remodeling, locally increasing the diffusion of damaged chromatin. While this may facilitate the search for broken ends and homologous rejoining, it may also be deleterious in the presence of a large amount of DSBs, triggering misrepair and chromosome rearrangement [43]. As reviewed in [44], extensive evidence supports the hypothesis that proximity between DNA breaks favors chromosomal translocation. Live imaging has shown that, while some breaks can travel over large distances, the majority (90%) of translocations are formed by proximal DSBs [45]. Consequently, many computational models of DNA damage and repair consider that the probability for two free ends to (mis)rejoin depends on the distance separating two breaks, known as the proximity effect [34,46,47,48,49,50]. As the spatial organization of chromosomes depends on the cell nucleus geometry, it is thus expected that the spatial organization of breaks, and in particular the distance from one another, will also be influenced by the geometry. The role of the nuclear geometry and more generally of the 3-dimensional (3D) genomic organization on the formation of CAs remains to be understood [44].

In this work, we examine how the volume and shape of nuclei, the orientation of the radiation beam for non-spherical nuclei, and the chromosome organization between domains (chromosome intermingling), influence the formation of CAs for mono-energetic ion beams of LET varying from 0.22 keV/μm up to 195 keV/μm. To that end, we used the Monte Carlo simulation codes Relativistic Ion Tracks (RITRACKS) and Radiation-Induced Tracks, Chromosome Aberrations, Repair and Damage (RITCARD) [50,51,52] to perform track structure calculations and model chromosome distribution within the nucleus, allowing us to predict CAs for a variety of nuclear geometries. We investigated the effect of nucleus size by considering spherical nuclei of radii varying from 2 μm to 8 μm. We also assessed the effect of the nucleus shape by fixing the volume of an ellipsoidal nucleus and decreasing the axis along the orientation of the irradiation beam while increasing the other two axes, as well as varying the beam orientation. We investigated the effect of cell size variability within a population on CA predictions. Finally, as experimental work showed that intermingling correlated with increased chromosomal translocation [38], this was investigated by constraining or not constraining chromosomes within non-overlapping domains to represent two extreme cases of intermingling.

## 2. Results

### 2.1. Nuclear Geometries Considered for This Work

**Figure 1:**
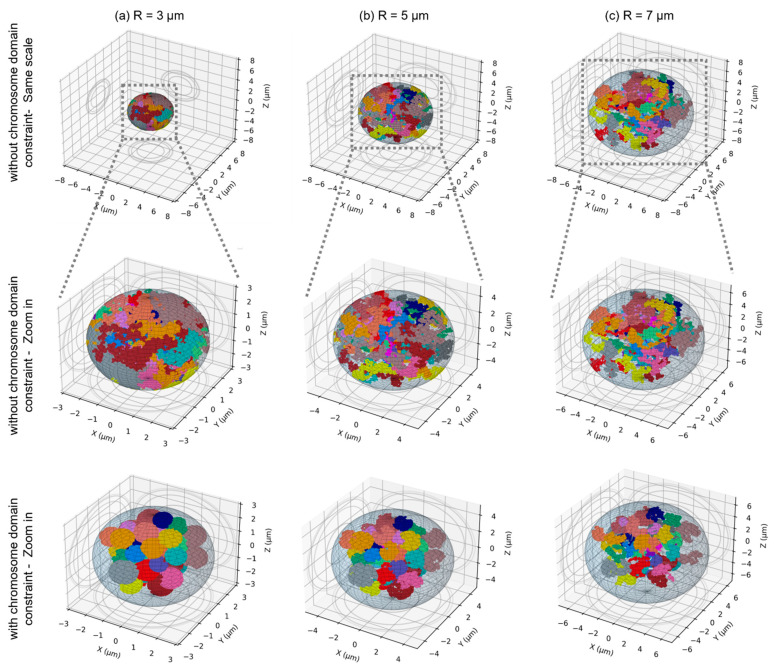
3D distribution of the 46 chromosomes within spherical nuclei of different sizes: (**a**) R = 3 µm, (**b**) R = 5 µm and (**c**) R = 7 µm. In the top row, the nuclei are represented at the same scale, and the chromosomes are not constrained within their domain. The same nuclei are displayed in the middle row with a magnification of each to visualize the different packing densities. In the bottom row, the chromosomes are constrained to be within their domain.

**Figure 2:**
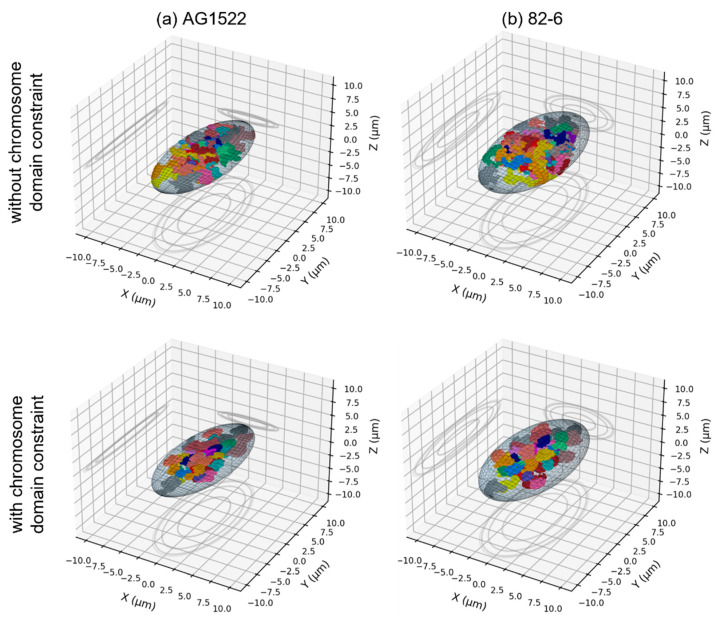
3D distribution of the 46 chromosomes within ellipsoidal nuclei representing (**a**) AG1522 human fibroblast [32] and (**b**) 82-6 fibroblast [53,54] grown on a substrate (adherent cell). In the top row, the chromosomes are not constrained to be within their domain, while they are in the bottom row.

**Figure 3:**
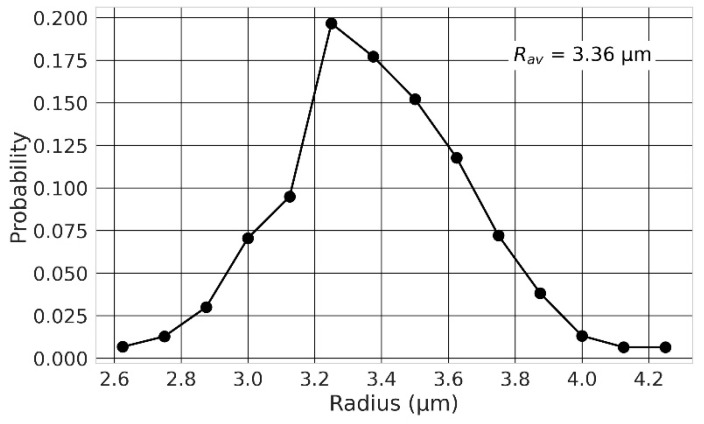
Probability distribution of lymphocyte nuclear radii. Adapted with permission from Ref. [26] © 2006, Elsevier. The average nuclear radius is 3.36 µm assuming a spherical distribution.

A random walk algorithm was applied to model the distribution of the chromosomes within the nucleus. Each chromosome consisted of a sequence of cubical monomers of length equal to 20 nm (2000 DNA base-pairs) and was associated with chromosome domain that scaled with nuclear dimensions and did not overlap with other chromosome domains (see Section 4.2 for more details). The parameters of the nuclear geometries are provided in Table 1. To investigate the effect of the nuclear size, we considered spherical nuclei of radii varying from 2 µm to 8 µm. A reduction in the nucleus volume induces a visible compaction of chromosomes (Figure 1). Additionally, we considered the effect of chromosome intermingling by sampling the position of the first monomer of each chromosome in their chromosome domain and constraining or not constraining the rest of the monomers within their (non-overlapping) domain. As shown of Figure 1, not constraining the chromosomes within their domain induces significant intermingling of the chromosomes that is particularly visible for small nuclei. We also investigated how a change from a spherical to an ellipsoidal shape impacted the results. To that end, we considered the size of two fibroblasts with ellipsoidal nuclei (AG1522 [32] and 82-6 [53,54]) (Figure 2) and modified the nucleus thickness along the irradiation axis while preserving the nucleus volume. We also considered the effect of the beam orientation by fixing the semi-axes of the nucleus and changing the beam orientation. Lastly, as the size of the nucleus can vary within a cell population, we evaluated whether dose-responses obtained for a distribution of nuclear sizes, as opposed to average size, are the same. To that end, we modelled a spherical lymphocyte and used the distribution of radii provided by [26] (Figure 3). We modelled 14 nuclei of radii varying from 2.63 µm to 4.25 µm and calculated the dose-response for each of them. To obtain the final dose-response of a cell population with the radius distribution of Figure 3, we summed, for each dose-response datapoint, the CA yields for all radii, weighted by their frequency. The dose-response was compared to the dose-response obtained for the average nucleus.

### 2.2. Effect of the Nuclear Volume

**Figure 4:**
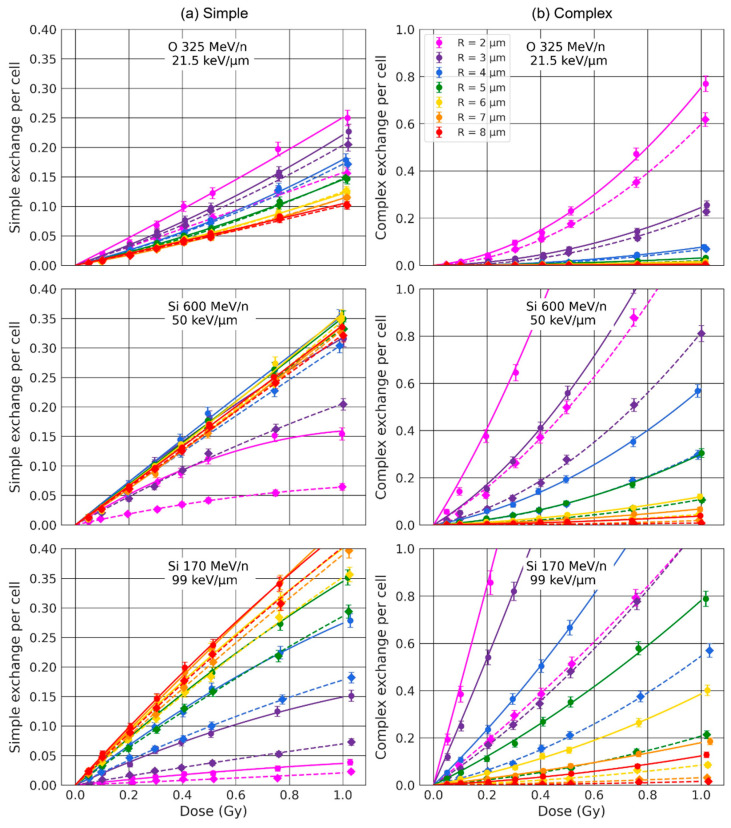
Dose-response of simple (**a**) and complex (**b**) exchanges for 3 ion beams and spherical nuclei of radii varying from 2 μm to 8 μm. The data points are calculated by RITCARD. The lines represent the best linear quadric fit of the dose-response. Solid line with round symbols: without chromosome domain constraint. Dashed line with diamond symbols: with chromosome domain constraint.

**Figure 5:**
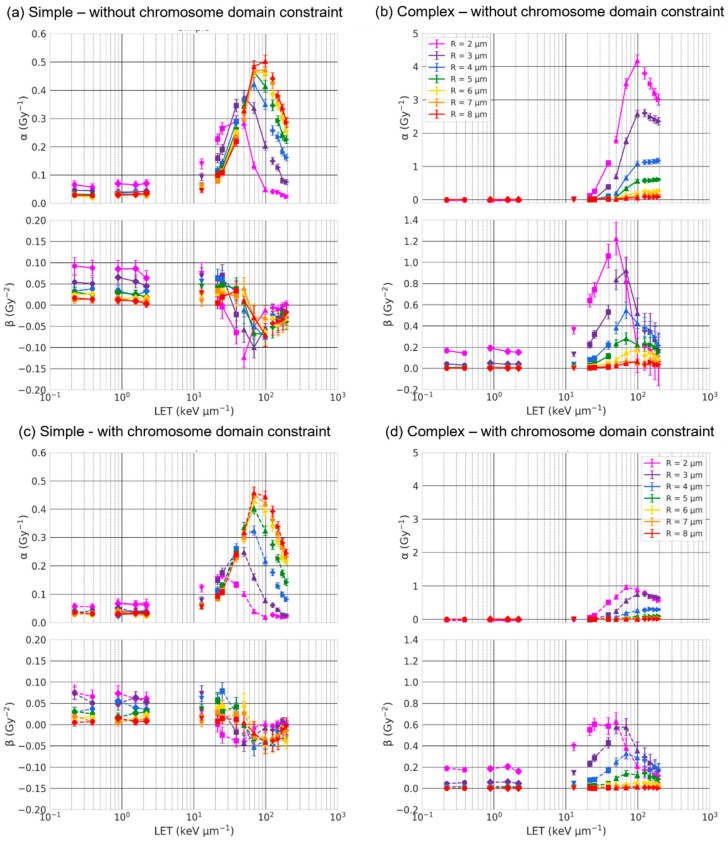
α and β coefficients of simple (**a**,**c**) and complex (**b**,**d**) exchanges as a function of LET and for spherical nuclei of radii varying from 2 μm to 8 μm. Solid (without chromosome domain constraint (**a**,**b**)) and dashed lines (with chromosome domain constraint (**c**,**d**)) are plotted as straight lines between RITCARD datapoints for a given ion type. Circle: H (1000 MeV, 250 MeV); Diamond: He (1000 MeV/n, 250 MeV/n, 150 MeV/n); Triangle down: C (290 MeV/n); Square: O (325 MeV/n, 250 MeV/n, 128 MeV/n); Triangle up: Si (600 MeV/n, 300 MeV/n, 170 MeV/n); Plus: Ti (600 MeV/n); Triangle right: Fe (1000 MeV/n, 600 MeV/n, 450 MeV/n).

**Figure 6:**
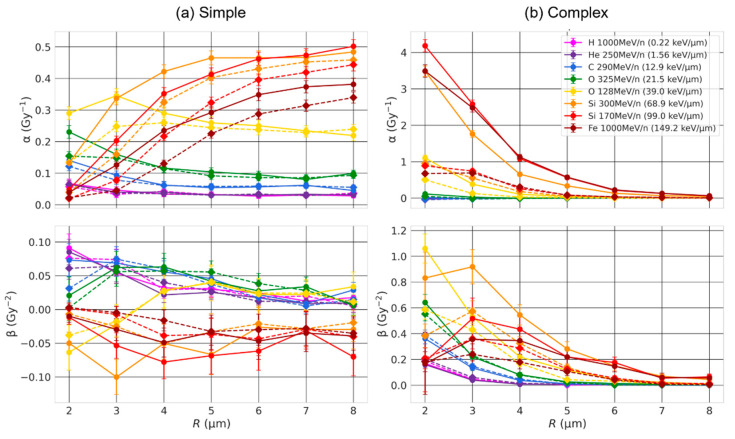
α and β coefficients of simple (**a**) and complex (**b**) exchanges as a function of the nuclear radius and for different given ion beams. Straight lines were added between RITCARD datapoints to help visualization. Solid line with round symbols: without chromosome domain constraint. Dashed line with diamond symbols: with chromosome domain constraint.

We first investigated how the nuclear volume impacts simple and complex exchanges by considering spherical nuclei of radius R varying from 2 μm to 8 μm. Dose–response curves are shown in Figure 4 for three ion beams of LET varying from 21.5 keV/μm to 99 keV/μm. The results are displayed as a solid line when chromosomes were not constrained within the non-overlapping domains (intermingling allowed) and as a dashed line when they were (no intermingling allowed). As shown, the size of the nucleus strongly influences the formation of both simple and complex exchanges. The yield of complex exchanges is systematically higher for smaller radii regardless of the dose and for all ions and LETs, while the yield of simple exchanges is higher for smaller radii for oxygen at 325 MeV/n and becomes lower for smaller radii when the LET increases. Constraining chromosomes within non-overlapping domains (dashed line) systematically reduces the yield of both simple and complex exchanges, regardless of the value of the beam LET. These results are confirmed by the graphs in Figure 5 and Figure 6, which show the α and β coefficients as a function of the LET (Figure 5) or the nuclear radius (Figure 6), with chromosomes constrained or not constrained in non-overlapping chromosome domains.

For complex exchanges, we can see that both α and β show a strong dependence on the radius, especially for high-LET (>~40 keV/μm). Except for very high-LET (>~70 keV/μm), α and β both monotonically decrease when the radius of the nucleus increases, indicating that complex exchanges are less prevalent in large nuclei. When the LET reaches ~70 keV/μm, we see a drop in the value of β for R<3 μm. We also observe that α and β have a trend as a function of the LET that has similar shapes when the nuclear radius increases, with mostly differences in intensities. Of interest, α reaches a peak (R ≤3 μm) or a plateau value (R>3 μm) when the LET is about 100 keV/μm. The same trends are obtained whether or not chromosomes are constrained within the domains, but with systematically lower values of both α and β when the chromosomes are not overlapping.

For simple exchanges, while β depends on the LET, it does not show a strong dependence on the nuclear radius nor chromosome intermingling, as shown in Figure 6. However, α varies with R in a way that depends on the LET. When the LET is below 39 keV/μm, we observe a mild decrease in α values with increasing R. The trend is reversed for higher LET values, and we can see that α increases with increasing R and reaches a plateau value for R=6 μm. As shown in Figure 5, α has the same peaked distribution as a function of the LET, regardless of the nuclear size. However, when the radius decreases, the maximum value of α is reduced and occurs at smaller LET values. For instance, when chromosome overlapping is allowed, the peak value is equal to 0.3 Gy^−1^ for LET ~40 keV/μm and R = 2 μm and increases to 0.5 Gy^−1^ for LET ~100 keV/μm and R = 8 μm. As for complex exchanges, we observe similar trends when the chromosomes are constrained in non-overlapping territories, but with smaller α values, which is more pronounced for smaller radii.

### 2.3. Effect of the Nuclear Shape

Next, we investigated how changes in nuclear shape might affect the rate of simple and complex exchange formation. We considered two cell lines (AG1522 and 82-6) with distinct cellular volumes and investigated how the flattening of the cell affects CA yields by changing the shape of the cell from a sphere to a flattened ellipsoid, as described in Table 1. Ion beams were oriented along the shortest axis to mimic an in vitro experiment.

**Figure 7:**
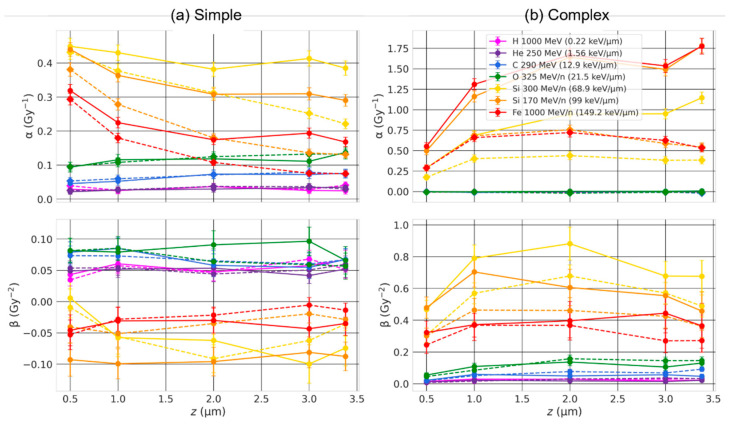
α and β coefficients of simple (**a**) and complex (**b**) exchanges as a function of the cell thickness, for AG1522 cell line (V = 162 µm^3^), and for ion beams of varying LET. Straight lines were added between RITCARD datapoints to help visualization. Solid line with round symbols: without chromosome domain constraint. Dashed line with diamond symbols: with chromosome domain constraint.

**Figure 8:**
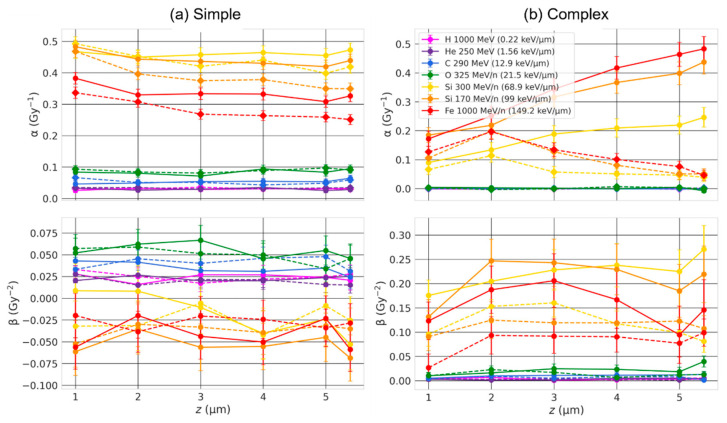
α and β coefficients of simple (**a**) and complex (**b**) exchanges as a function of the cell thickness, for 82-6 cell line (V = 656 µm^3^), and for ion beams of varying LET. Straight lines were added between RITCARD datapoints to help visualization. Solid line with round symbols: without chromosome domain constraint. Dashed line with diamond symbols: with chromosome domain constraint.

The coefficients α and β are shown in Figure 7 and Figure 8 for simple and complex exchanges as a function of the cell thickness and for ion beam of LET varying from 0.22 keV/µm to 149.2 keV/µm. As expected, the coefficients (especially α) depend both on ion LET and cell type, with overall smaller coefficients for the larger cell line (82-6) consistent with what we observed in the previous section. For simple exchanges, we can see little dependence on the cell thickness for low-LET beams but a marked dependence for beams of LET ≥ 68.9 keV/µm, with a small but significant decrease in α as the cell thickness increases especially for AG1522 (smaller volume), whether chromosomes intermingle or not. Regarding complex exchanges, we observe either small differences on the cell thickness (low-LET) or an increase in α values (high-LET) for increasing thickness when the chromosomes overlap (solid line), and an increase, followed by a decrease when they do not overlap (dashed).

### 2.4. Effect of the Beam Orientation

**Figure 9:**
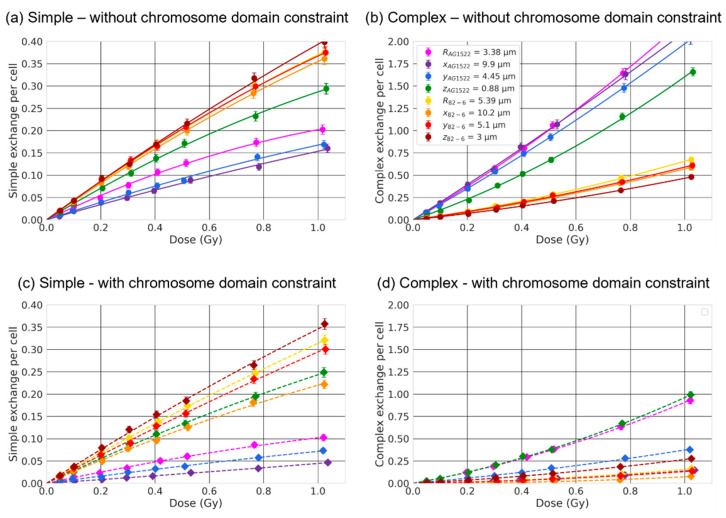
Simple (**a**,**c**) and complex (**b**,**d**) exchange dose-responses for two fibroblast cell lines (AG1522 and 82-6) irradiated by a 170 MeV/n Si ion beam. The data points are calculated by RITCARD. The lines represent the best linear quadric fit of the dose-response. The cell line was modeled either as a sphere ($R_{AG1522}=$ 3.38 µm and $R_{82-6}=$ 5.39 µm) or an ellipsoid of equivalent volume and irradiated along the different semi-axes x, y, or z. Solid line with round symbols (**a**,**c**): without chromosome domain constraint. Dashed line with diamond symbols (**b**,**d**): with chromosome domain constraint.

Next, we investigated how the beam orientation impacted the formation of CA for fixed nuclear geometries. The number of simple (a,c) and complex (b,d) exchanges are shown in Figure 9 as a function of the dose for spherical or ellipsoidal AG1522 and 82-6 fibroblast nuclei. Results are shown with ((c) + (d)) or without ((a) + (b)) chromosomes constrained within non-overlapping chromosome domains. The effect of the beam orientation was investigated by irradiating the nucleus with a 170 MeV/n Si ion beam along the different axes, noted as x, y and z (x>y>z) for the ellipsoidal shape, and R for the spherical shape. Values of the α and β coefficients are provided in Table 2.

The orientation of the beam influences the number of both simple and complex exchanges, and the effect is stronger for smaller nuclear volumes (AG1522). Such results are consistent with the fact that simple and complex exchanges reach a limit for large radii and therefore show little dependence on the geometry for large nuclei (Figure 6).

For simple exchanges, we can observe that yields increase when irradiating along shortest axis, regardless of the nuclear size or chromosome domain constraints. For complex exchanges, the trend is more complex and depends on whether the chromosomes are constrained within non-overlapping domains or not. In the case where chromosomes overlap, we see an increase in the yield when irradiating along the shortest axis for AG1522 and very similar yields for 82-6. When chromosomes do not overlap, we see the opposite trend for AG1522 and 82-6, with increased yields when irradiating along the longer axes.

### 2.5. Effect of the Nuclear Size Variability

Last, as several authors reported that the volume of the nucleus can differ within a cell population [26,30], we investigated the effect of considering a distribution of nuclear sizes as opposed to an average size. We evaluated CA yields for a distribution of spherical lymphocytes, of radii varying from 2.63 µm to 4.25 µm, based on the nuclear size distribution provided by [26] (Figure 3). This distribution reaches an average nuclear radius of 3.36 µm. The radius distribution encompasses relatively small radii, for which there exists a strong CA dependence, especially when chromosomes can overlap. We simulated the irradiation of lymphocytes by 170 MeV/n Si ion beams with overlapping chromosomes, as it was among the conditions that showed the strongest dependence on the nuclear radius (Figure 6). Altogether, we expected to maximize possible differences between predictions for a nuclear size distribution as opposed to an average radius.

**Figure 10:**
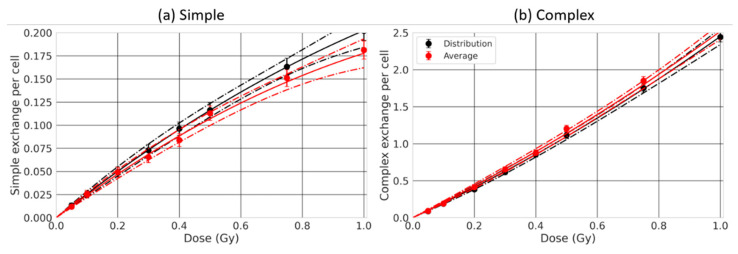
Simple (**a**) and complex (**b**) exchange dose-responses for a simulated irradiation by 170 MeV/n Si ion beam, considering a distribution of lymphocyte radii (black) or an average lymphocyte radius (red). The datapoints are results obtained by RITCARD, the solid line represents the best linear quadratic fit of the dose-response, and the dashed lines represent the 95% confidence interval.

The dose-response for simple (left) and complex (right) exchanges is shown in Figure 10, considering an average nuclear radius size (red) or a distribution (black). We can see that the two dose-responses are close.

## 3. Discussion

As listed in Table 3, cells have distinct nuclear shapes and volumes, with volumes ranging from 11.5 µm^3^ up to 1010 µm^3^. As the 3D chromosome arrangement depends on nuclear geometrical properties [25,36,37], we investigated the effect of nuclear shape and size on CAs, focusing on simple and complex exchanges.

### 3.1. Effect of the Nuclear Volume

Our first major result shows that the number of both simple and complex exchanges is heavily influenced by the size of the nucleus in a way that is LET-dependent (Figure 4, Figure 5 and Figure 6). Nuclei contain a fixed amount of DNA (~6 Gbp during interphase) organized in 46 chromosomes located within chromosome territories. Reducing the nuclear volume therefore changes the nuclear DNA density. For instance, Dos Santos et al. [27] estimated with Geant4-DNA simulations that DNA occupies 0.42% of the nuclear volume for fibroblast and 1.43% for endothelial cells. In our model, the chromosome domains have a volume that scales with the nuclear volume. Chromosome distribution is therefore influenced by nuclear geometrical properties. As shown in Figure 1, small nuclei appear to have a more compact DNA and quite significant intermingling when chromosomes are not constrained within non-overlapping domains, favoring chromosome exchanges [38]. When nuclei become larger, chromosomes appear more spaced out as the domains become larger, and we can see that less intermingling occurs. The distributions appear more qualitatively similar whether chromosomes are constrained in the domains or not. Such differences in distribution impact the yield of CAs.

**Figure 11:**
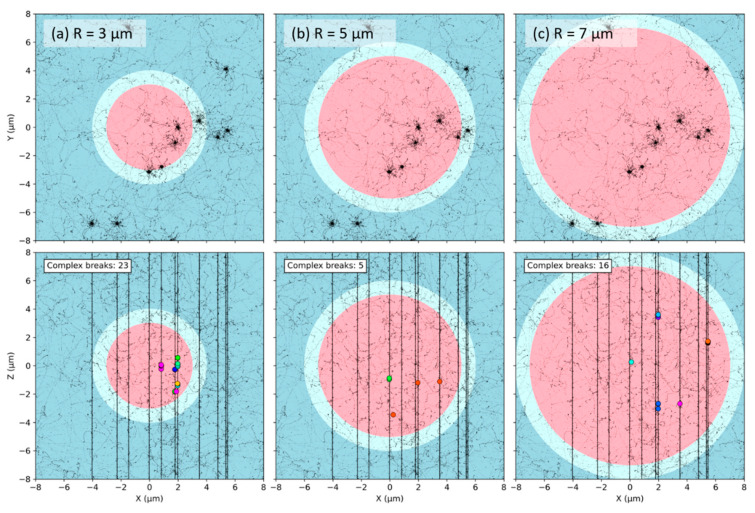
Projected track structure (black dots and lines) and complex DSBs (colored circles) before repair for a Si 170 MeV/n Si irradiation (LET 99 keV/µm) and dose of 0.5 Gy. Views are shown from atop (top) and side (bottom). The disks represent the cell nucleus (pink) and cytoplasm (cyan), embedded in water (blue). Results are shown for three nuclear radii ((**a**) 3 µm, (**b**) 5 µm, and (**c**) 7 µm) and without chromosome domain constraint. The different colors of the DSBs represent distinct chromosomes.

At the nanometric (DNA) scale, simulations of early DNA damage showed that the complexity, defined as the number of clustered damages on DNA strands that could lead to DSBs, was always higher for endothelial cell nuclei as compared to fibroblast nuclei [27]. In our model though, the number of DSBs does not depend on geometrical properties and is about 35 DSB/Gy/cell. On a larger, micrometric (nucleus) scale, the volume of the nucleus impacts the distribution of chromosomes (Figure 1) and consequently, the distribution of complex DSBs (i.e., breaks involved in misrepairs and thus CA formation in our model, see Section 4.4 for more details). Figure 11 represent the distribution of complex DSBs for three nuclear radii, using the same track-structure history for a 0.5 Gy Si 170 MeV/n irradiation. The black lines (ion cores) are made of thousands of points, each representing an ionization or excitation event. The smaller black dots visible across the whole volume are ionization and excitation events due to δ-electrons. Due to the stochastic nature of the simulations, we expect a great variability in break formation depending on the simulation history; however, we show here examples that we expect to be representative of the effect of nuclear volume variation. Note that, for a given dose, the number of DSBs per cell follows a Poisson distribution (Equation (3)), with an average number that does not depend on the cellular geometry, as the DNA amount is kept constant. However, if we averaged the number of breaks for a large cell population, we would find ~17.5 DSBs/cell regardless of the nuclear radius. In these three examples, we obtain a total number of complex DSBs that is equal to 23, 5, and 16 for the cells of radii equal to 3 µm, 5 µm, and 7 µm respectively. We observe that as the size of the nucleus increases, the complex DSBs become more spaced out and less clustered together. On the contrary, small nuclei have complex DSBs that appear clustered and close together. Such observations are the result of chromatin fibers being less compact and more spread out for larger nuclei (Figure 1). Since the probability of misrejoining depends on the Euclidian distance between breaks in our model, small nuclei favor the formation of misrejoining, leading to an increase in both simple (LET < 40 keV/µm) and complex exchanges (regardless of the LET) when the radius decreases. Simple exchanges start decreasing for high-LET when the nuclear volume decreases, likely because as the LET increases, the number of track traversals considerably decreases (see Table 1). As the number of average nuclear track traversals gets close to or even below 1, the number of chromosomes crossed by tracks get smaller, and the DSBs get clustered. For one track traversal, smaller nuclei increase the chances of crossing more chromosomes as they are closer together. Altogether, this likely favors complex over simple exchanges for small nuclei and explains why, for high-LET, we observe an increase in the number of simple exchanges for larger nuclei while a majority of exchanges in small nuclei are of complex nature. The trends are similar whether the chromosomes intermingle or not.

As nuclei can vary in size within a cell population, we also evaluated the effect of a distribution of cell nuclei, as compared to an average size, on CA yields. Using an experimental lymphocyte distribution reported by Loiko et al. [26] and an ion beam (Si 170 MeV/n) that would maximize differences, and chromosome intermingling allowed, we obtained similar results when comparing the two predictions. Within the range of radii considered for this distribution, both α and β coefficients show a quasi-linear dependency with the radius (Figure 6). Combined with a quasi-normal radius distribution, it is not surprising that the two dose-responses are close. Other ion beams, which show similar or weaker α and β coefficient dependencies on the radius, are expected to show similar trends. This suggests that considering an average volume, as long as the distribution is not too wide and close to a normal distribution, is a good approximation to represent a cell population during interphase. A more skewed size distribution over a wider radius range would likely show more differences as the dependency on the radius would no longer be quasi-linear. Change in cellular shape within cell population, which we did not test, could also affect dose-responses more significantly, but experimental data with such distributions are needed.

**Figure 12:**
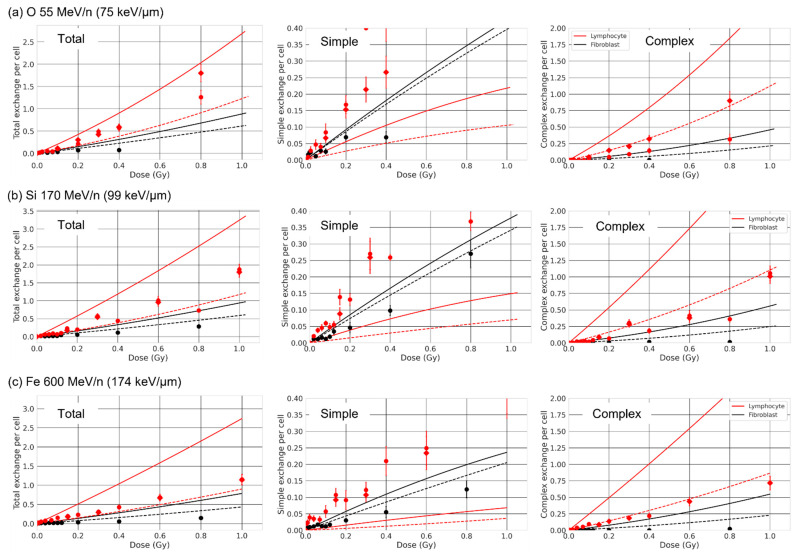
Comparison between experimental results and RITCARD predictions for lymphocytes (red, spherical with R = 3 µm) and fibroblasts 82-6 (black, ellipsoidal with x=y= 7.22 µm and z= 3 µm) for ion beams (**a**) O 55 MeV/n, (**b**) Si 170 MeV/n and (**c**) Fe 600 MeV/n. Left to right: total, simple, and complex exchanges. The solid lines (without chromosome domain constraint) and dashed lines (with chromosome domain constraint) are the LQ fit of the results from RITCARD. Datapoints are experimental results adapted from Ref. [68] (circles) and Ref. [21] (diamonds) © 2022 Radiation Research Society. Cell sex is not reported in the studies.

We compared our results with experimental results reported by Hada et al. [68] and George et al. [21]. The authors measured simple and complex exchange dose responses for O 55 MeV/n, Si 170 MeV/n and Fe 600 MeV/n, for lymphocytes (spherical nucleus with a radius ~3 µm) and 82-6 fibroblasts (ellipsoidal nucleus, semi-axes of ~7.22 µm and thickness we set to 3 µm). They find that complex exchanges are systematically higher for lymphocytes (small nucleus) than fibroblasts (large nucleus), consistent with the trend we obtain. Similar observations were obtained in Loucas et al. [55], where the authors show a higher number of complex exchanges for lymphocytes compared to fibroblasts (AG1521) for γ-ray irradiation. A comparison with our predictions (Figure 12) shows that we obtain a number of complex exchanges systematically higher than experimental results when intermingling is allowed, especially for lymphocytes, and closer to experimental results when there is no intermingling. In the former case, the amount of intermingling is likely largely overestimated especially for small nuclei. A geometry with a much more restricted overlapping of chromosomes is likely to be more realistic. Additionally, Hada and colleagues report a systematic higher number of simple exchanges for lymphocytes compared to 82-6 fibroblasts, while we observe a reversed trend for these LET values. Several reasons could account for these differences. Our repair model has repair time constants that are based on experimental results for fibroblasts and may need to be adjusted for lymphocytes. Lymphocytes are also typically allowed to repair for longer than 24 h, while we modeled repair up to 24 h. Besides, our CA classification categorizes complex exchanges as CAs involving more than two breaks, with two or more chromosomes, regardless of the fragment length. In [68], the authors used the fluorescent in situ hybridization (FISH) technique with a limit of detection reported to be typically equal to ~10 Mbp [69]. Not considering such detection threshold could, in turn, yield higher complex exchanges in a way that might depend both on the radiation quality and the size of the nucleus, as high-LET ions are known for inducing very small fragments. Additionally, Hada et al. [68] used the FISH technique to stain chromosomes 1, 2, and 4 and applied a whole genome equivalent formula, while we consider the whole genome. Some exchanges could be experimentally labeled as simple while we would obtain complex exchanges. Looking at total exchanges (Figure 12), we obtain the same trend when comparing with experimental measurements for both fibroblasts and lymphocytes but with a systematic higher yield. It is possible that the detection limit of 10 Mbp would reduce this yield, and a partial staining of the whole genome would modify the balance between simple and complex exchanges. Finally, we also count exchanges for every cell 24 h after irradiation. Experimentally, it is possible that the most damaged cells (i.e., containing a large number of complex exchanges) would not survive or might not arrive at mitosis and thus would not be counted, although premature chromatin condensation technique should limit such bias [18,54,68]. We plan, in the future, to investigate how experimental detection limits and partial staining of the genome impact the yields we calculate with RITCARD.

### 3.2. Effect of the Nuclear Shape

**Figure 13:**
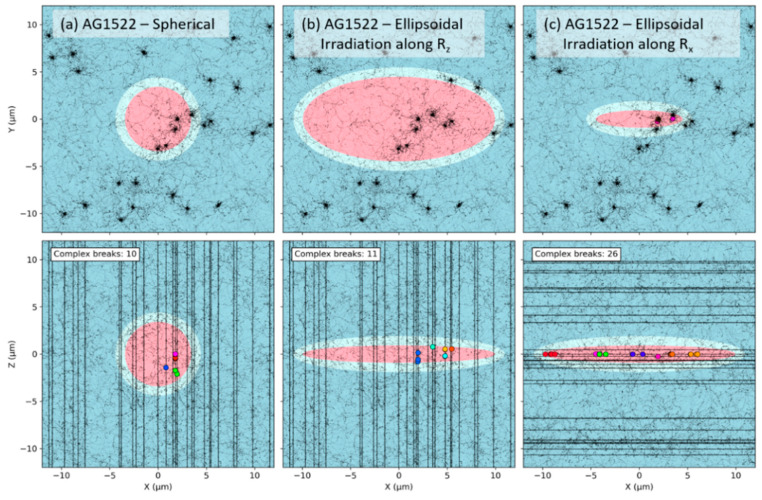
Projected track structure (black dots and lines) and complex DSBs (colored circles) before repair, for a Si 170 MeV/n Si irradiation (LET 99 keV/µm) and dose of 0.5 Gy. Views are shown from atop the beam line (top) and side (bottom). The disks represent the cell nucleus (pink) and cytoplasm (cyan), embedded in water (blue). Results are shown for the AG1522 fibroblast (V = 162 µm^3^) and without chromosome domain constraint, for (**a**) a spherical shape, and an ellipsoidal shape irradiated along (**b**) the thinner axis, and (**c**) along the longest axis. The different colors of the DSBs represent distinct chromosomes.

Ingram et al. [36] experimentally measured genome contact maps (Chromosome Conformation capture data (Hi-C)) of different cell types to reconstruct 3D nuclear geometries and coupled the nuclear geometries with the track-structure code Geant4-DNA to calculate the DNA DSB distribution. They investigated the effect of nuclear shapes by simulating spherical and ellipsoidal nuclei. They studied DNA DSB spatial distribution by applying a cluster algorithm, i.e., counting the number of DSBs located within a sphere of a given radius. As expected, the difference in nuclear geometries did not impact the yield of DSBs per cell, as the amount of DNA was fixed (6 Gbp). However, they obtained significant differences in DSB clustering. They concluded that change in nuclear shapes likely impacts CA yields, which is what our calculations show. The nuclear shape influences the formation of both simple and complex exchanges, with yields depending on the thickness of ellipsoidal nuclei (Figure 7 and Figure 8). The change in nuclear shape affects the distribution of complex breaks, as shown in Figure 13. As the nucleus is flattened, the number of track traversal increases, but the thickness that is traversed decreases. Consequently, the complex breaks are spread out across the nucleus irradiation area, while the number of DSBs along one track tend to decrease, which likely explains why, overall, we usually see an increase in complex exchanges when the thickness increases.

A few experimental studies investigated the effect of nuclear shape on CAs. Schmid et al. [33] considered lymphocytes that were either settled or attached, thus having either spherical or flattened nuclei, with vertically traversed diameters of 5.5 μm (spherical) and 3.0 μm (flattened). They focused on *F* values, defined as the ratio of dicentrics to centric rings. Dicentrics are interchromosomal exchange-type aberrations, thus involving two chromosomes, while centric rings are intrachromosomal, interarm exchange-type aberrations, thus involving only one chromosome. Using ^241^Am α particles (most probable energy of 2.7 MeV with an LET of 150 KeV/μm) and doses of 0.1 Gy and 1 Gy, they obtained a higher number of centric rings (intrachromosomal) and dicentrics (interchromosomal) for settled cells (sphere), compared to attached cells (ellipsoid), regardless of the dose. *F* values were equal to 4.27 (±0.44) vs. 10.07 (±1.73) for settled (spherical) vs. attached (flattened) cells. Such results are consistent with the fact that flattened nuclei have chromosome territories that are dispersed horizontally, leading to fewer chromosome territories traversals per tracks. As α particles have a very high LET, DNA damages are mostly localized along the track, which in turn might reduce breaks proximity from distinct chromosomes when nuclei are flat, thus favoring intrachromosomal rearrangements and reducing chances of having complex exchanges, which is what we observe in Figure 7 and Figure 8 if chromosomes intermingle.

Another group of studies [4,35] also investigated the effect of nuclear shape by looking at the ratio of simple over complex exchanges (s:c) for various cell lines irradiated by ~1 perpendicular ^238^Pu α-particle (LET of 121 keV/μm) using a multicolor FISH approach. They obtained an s:c ratio of ~2 for flattened normal human bronchial epithelial (NHBE) cells, as opposed to <1 for spherical peripheral blood lymphocytes or hematopoietic stem cells. One possible explanation for such differences was attributed to the flat geometry of the cell nucleus, as opposed to a spherical one for lymphocytes and haemopoietic stem cells, and thus the difference in chromosome territories organization. To compare such ratios with our data, we extrapolated the number of simple and complex exchanges for an average track traversal of 1 and calculated the exchange s:c ratio for the cellular geometries investigated. Results are for Si 170 MeV/n irradiation (LET of 99 keV/μm), although similar trends are obtained for Fe 1000 MeV/n irradiation (LET of 149.2 keV/µm). We observe a decrease in the s:c ratio from 2.55 (1 μm in half-thickness) to 0.90 (spherical with 5.39 μm radius) for 82-6, and from 0.83 (0.5 μm in half-thickness) to 0.13 (spherical with 3.38 μm radius) for AG1522 when chromosomes intermingle. When chromosomes do not overlap, we still observe a decrease in the s:c ratio from 1.25 (0.5 μm in half-thickness) to 0.17 (spherical with 3.38 μm radius) for AG1522, while it first decreases from 4.21 (0.5 μm in half-thickness) to 2.70 (3 µm in half-thickness) before increasing again and reaching a ratio of 4.95 (spherical with 5.39 μm radius). With one exception, we thus observe that round nuclei favor complex over simple exchanges for one track traversal.

### 3.3. Effect of the Beam Orientation

As the shape of the nucleus impacts CA yields due to differences in the number of traversed chromosomes, we also investigated how the beam orientation, for a fixed ellipsoidal geometry, influences the number of CAs by simulating irradiation along the three axes x>y>z. We obtained distinct yields with, in particular, simple exchange yields higher when irradiating along shorter axes. Such observations are also related to the distribution of complex DSBs, as shown in Figure 13. When going from an irradiation along the shortest axis to one along the longest axis, we see a reduction in the number of track traversal but also an important cluster of DSBs along the tracks that do traverse in the latter case.

Durante et al. [32] investigated the effect of beam orientation by irradiating ellipsoidal fibroblasts (AG1522) along the different nuclear axes with a 1000 MeV/n Fe beam (LET = 145 keV/μm). A mFISH analysis (whole genome) was performed to compare simple over complex exchange yields. For a dose of 1 Gy, they showed that the number of CAs and their complexity depended on the beam orientation. They obtained an increasing number of simple exchanges when irradiating along shorter axes, consistent with what we obtain. However, they obtained the highest number of complex exchanges when irradiating along the y axis, while we obtained the highest number of complex exchanges when irradiating along the x axis (without chromosome domain constraint) or the z axis (with chromosome domain constraint).

### 3.4. Effect of Chromosome Intermingling

As reviewed in [39], while a few studies suggest that chromosome intermingling is limited, many studies have shown that there is extensive intermingling between neighboring chromosomes. For instance, Branco et al. [38] reported that 41% of the volume of chromosome 3 intermingles with the remaining genome. The amount of intermingling was reported to depend on the cell line or the cell shape [36,39]. It was also suggested that the amount of intermingling correlates with increased chromosomal translocation [38]. To study this effect, we generated two distinct chromosome distributions for a fixed nuclear geometry. In both cases, the nuclei had non-overlapping chromosome domains of fixed position, and the position of the first monomer of each chromosome was randomly sampled inside the associated chromosome domain. Then in one case, monomers of a given chromosome were constrained to be contained within the chromosome territory (no intermingling) while in the other case they were not, leading to intermingling that could be particularly important for small nuclear geometries. These two cases intend to represent two extreme cases that would bound the effect of intermingling. Note that it likely overestimates intermingling in the case of small nuclei, as intermingling is usually reported to occur at the edge of chromosome territories [39].

**Figure 14:**
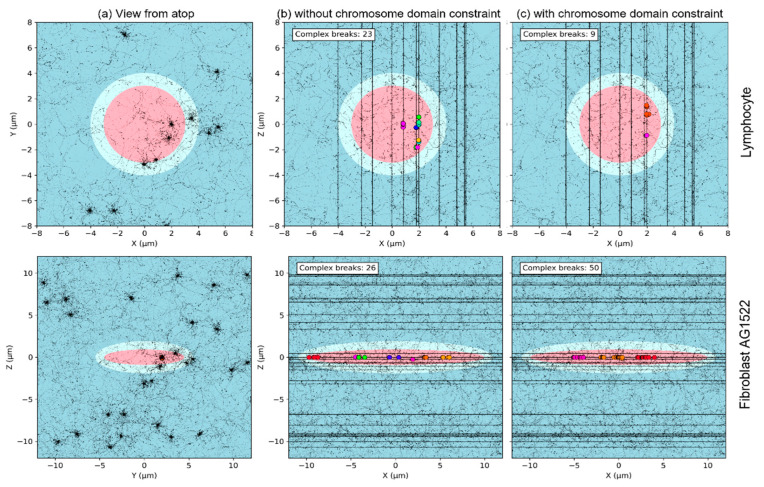
Projected track structure (black dots and lines) and complex DSBs (colored circles) before repair, for a Si 170 MeV/n Si irradiation (LET 99 keV/µm) and dose of 0.5 Gy. Views are shown from (**a**) atop the beam line and (**b**,**c**) side. The disks represent the cell nucleus (pink) and cytoplasm (cyan), embedded in water (blue). Results are shown for a spherical radius of radius equal to 3 µm (top) or an ellipsoidal AG1522 fibroblast, (**b**) without chromosome domain constraint and (**c**) with chromosome domain constraint. The different colors of the DSBs represent distinct chromosomes.

As results show, the major effect of chromosome intermingling is to increase the yields of both simple and complex exchanges in agreement with observations by Branco and colleagues [38], with a more significant increase for complex exchanges. This effect is predictably more pronounced for smaller nuclei. As chromosomes have a fixed length, decreasing the size of nuclei increases overlapping between chromosomes, as compared to larger nuclei. Interestingly, for very high LET beams and complex exchanges, we also observe that trends regarding the nuclear shape or beam orientation differ whether chromosomes intermingle or not. The effect of chromosome intermingling on complex DSB distributions is shown Figure 14 for two nuclei. As we can see, the major effect of intermingling is to change the number of chromosomes that are traversed for a given track and consequently the proximity between breaks of distinct chromosomes, which is particularly visible in Figure 14 for the case of the AG1522 fibroblast irradiated along the longest axis. When intermingling is allowed, we see a great variation of the break colors along the traversing tracks, meaning many chromosomes are broken along the tracks. The number of colors is greatly reduced when there is no intermingling, and we see clusters with breaks of the same chromosomes appearing along the track. As breaks from distinct chromosomes are not as clustered without intermingling, we thus see a large decrease in both simple and complex exchanges.

## 4. Materials and Methods

We used RITRACKS and RITCARD [50,51,52] to perform track structure calculations and model chromosome distribution within the nucleus, allowing us to predict CAs for a variety of nuclear geometries. RITCARD [50,52] is a chromosome aberration model based on the NASARTI (NASA Radiation Track Image) model developed by Ponomarev et al. [47,59,70,71]. NASARTI was originally developed to model DNA breaks using amorphous tracks and benchmarked against PFGE (pulsed-field gel electrophoresis) data [71]. It was further extended to add repair kinetics and chromosome aberration calculations [47,70], and provided reasonable agreement with experimental yields of simple/complex exchanges for photon and 1000 MeV/n Fe irradiation and various cell lines [70]. Significant improvements on NASARTI implementation led to a new software architecture, subsequently named RITCARD [50]. It notably relies on track structure simulations from RITRACKS [51] to calculate DSBs, contrary to NASARTI (amorphous track). RITCARD provided reasonable agreement with experimental simple exchanges for human normal fibroblasts (82-6) for ion LETs in the range 0.39–170 keV/µm [50], which was further improved by the implementation of a new repair algorithm [52]. This new algorithm could also reproduce experimental repair kinetics for ions over a large LET range for a time period up to 24 h (γ-H2AX immunostaining, human fibroblast), and fraction of unrepaired breaks at 3 h (gel electrophoresis, various cell lines). RITCARD also shows good agreement with the experimental yield of simple exchanges for fibroblasts irradiated by mixed fields [53].

**Figure 15:**
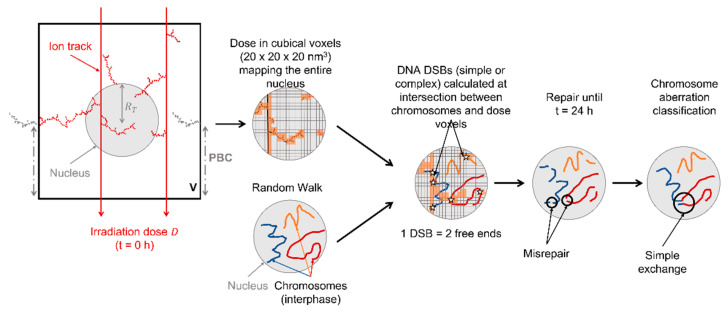
Two-dimensional representation of the different steps in RITRACKS/RITCARD for chromosome aberration calculations. DNA DSBs are defined as simple or complex based on an energy threshold cutoff of 500 eV within a given nanometric voxel. In our model, we assume that only complex DSBs can lead to misrepair. PBC: periodic boundary condition.

The different steps to calculate CAs with RITRACKS/RITCARD are illustrated in Figure 15. As part of RITRACKS, the first step consists of irradiating the water-filled cell nucleus with a given ion type and energy. As a second step, the dose in cubical voxels that map the entire nucleus is scored. In parallel, the chromosomes in the cell nucleus are modeled using a random walk with constraints [47,59,70]. In RITCARD, the next step consists of calculating intersection between chromosomes and voxels that have a dose larger than 0 Gy to calculate DNA DSBs. Then, an algorithm modeling repair over a 24 h time period during which free ends can be repaired or misrepaired is applied [52]. As a last step, chromosome aberrations are classified. The next sections detail the sequential steps.

### 4.1. Nucleus Irradiation

As illustrated in Figure 15, the first step consisted of simulating the nucleus irradiation to compute a 3D nanometric voxel dose map. We used the Monte Carlo track structure code RITRACKS [51] to model the transport of ions through liquid water, the main constituent of cells. The ions can be of various energies and atomic numbers, and the transport includes the cascade of secondary electrons produced in liquid water. Energy deposition events (ionizations/excitations) form the ion track, which is commonly described for high-LET ions as a dense ionization core and a penumbra made of low-LET δ-electrons.

In the present study, calculations were performed by defining a parallelepiped irradiation volume, V, that encompassed a spherical or ellipsoidal nucleus filled with water. The number of ions, n, crossing V for each simulation history was obtained by sampling a Poisson distribution,

(1) $$p\left(n\right)=\frac{\lambda^{n}\exp\left(-\lambda \right)}{n!}\;\;\;\;\;\;\mathrm{and}\;\;\;\;\;\;\;\;\lambda =\phi A.\;\;$$

λ is the average number of tracks traversing V, A is the surface of irradiation of V, and ϕ is the beam fluence obtained from the well-known equation,

(2) $$D\;\left(\mathrm{Gy}\right)=1.6\times 10^{9}\phi \;\left({\mathrm{cm}}^{-2}\right)\;\mathrm{LET}\;\left(\mathrm{keV}/\mu m\right).$$

In Equation (2), D is the irradiation dose, and the LET is obtained using Bethe’s equation with corrections [72]. The list of ion types and energies investigated in this study is provided in Table 4. Ions have a range in water spanning a few centimeters up to a few meters, well beyond the nuclear sizes investigated in this study.

For ions simulated in the present study, ejected δ-electrons have an energy distribution spanning a few eV up to hundreds of keV, with path lengths in tissue that can be well beyond a few millimeters. Simulating such large distances with RITRACKS would result in prohibitive calculation times. As an approximation, we applied periodic boundary conditions to account for more energetic δ-electrons that are produced by nearby ion tracks that may not have directly intersected the nucleus volume. As illustrated in Figure 15 by the grey part of the ion track, when a secondary particle leaves the irradiated volume, it appears on the opposite side with the same velocity vector. This approach ensures that the total dose deposited in the irradiated volume V matches the input irradiation dose, D, and that no energy is lost due to secondary particles travelling outside V. V is set large enough to avoid artifacts such as energetic δ-electrons crossing the volume an unrealistic number of times. These calculations showed that considering an irradiation area side length of 15 µm or larger was enough to avoid such artifacts for the nuclear geometries considered. More detailed analyses on the use of periodic boundary conditions in simulations of track structure and CA formation using RITRACKS/RITCARD can be found in [73,74].

### 4.2. Random Walk and Chromosome Geometries

In parallel to the simulation of the track structure with RITRACKS, a random walk algorithm is used and shown in Figure 15 to model the geometrical distribution of chromosomes within the nucleus. We considered a human male cell nucleus during interphase, thus containing a total of 46 chromosomes. In comparison, female nuclei have 1.7% more DNA content. We do not expect such increase would change the trends reported in this work and would likely yield very little differences in terms of absolute CA yields. The parameters of the genome (total chromosome length and centromere position) were taken from experimental measurements [75]. The chromatin fiber was approximated by a polymer chain: a chromosome was modelled by a sequence of monomers whose spatial geometry was obtained by a random walk on a cubic lattice [71]. One monomer represented 2 kbp of DNA and had a lattice period equal to 20 nm, corresponding to a linear density of 10 nm per 1 kbp. Each chromosome was made of sequences that consisted of a 60-monomer loop (120 kbp) followed by a 60-monomer linker. The loop was generated with a brute force approach by simulating a 60-monomer sequence until it closes onto itself [71]. Our current model does not consider intermediate levels of chromatin organization, such as open/closed compartments, topologically associated domains or lamina-associated domains [76].

The whole genome was constrained to be within the nucleus. Additionally, chromosomes were associated with non-overlapping spherical or ellipsoidal domains of fixed position, the size of which scaled both with the chromosome length and the size of the nucleus. Spherical domains were originally obtained for a spherical nucleus of radius equal to 6 µm, and domain radii were proportional to the chromosome size. When changing the size of the nucleus, the domain axes were then scaled with the nuclear axes. Chromosomes are contained within chromosome territories, but it has been shown that intermingling between different chromosomes occurs [38,39]. We thus tested two cases. In both cases, the position of the first monomer was randomly sampled in its chromosome domain, allowing for spacing out chromosomes within the nucleus. Then, in one case, the rest of the chromosome was contained within the chromosome domain. As the domains did not overlap, intermingling of the chromosomes was prevented. In the second case, the rest of the chromosome was not constrained in the domain, allowing it to intermingle with surrounding chromosomes. For each nucleus geometry, we generated only one chromosome distribution. Increasing the number of nucleus distributions to five, by executing the random walk five times with distinct seeds, yielded results that were within error bars. In our calculation, CA yields are not sensitive to the variation of chromosome distributions given a fixed nuclear geometry likely because the domains are fixed and the CA yields are obtained for the whole genome.

### 4.3. DNA Damage

To compute the 3D distribution of DSBs within the nucleus, the dose was scored in 20 × 20 × 20 nm^3^ voxels that mapped the cell nucleus using RITRACKS track structure outputs. The 3D dose map, together with the chromosome distribution obtained by the random walk, was then used to compute DNA DSBs by locating intersections between chromosomes and voxels for which the dose was higher than 0 Gy. The number of DSBs, N contained in a monomer was then given by the Poisson distribution,

(3) $$p\left(N\right)=\frac{\lambda^{N}\exp\left(-\lambda \right)}{N!}\;\;\;\;\;\;\mathrm{and}\;\;\;\;\;\;\;\;\lambda =Q\cdot D\left(i,j,k\right),$$

where D(i,j,k) is the dose in the voxel of spatial coordinates (i,j,k) in lattice units, and Q is an adjustable parameter representative of the intensity of DSB formation per unit dose. PFGE experiments show that this parameter has a weak dependence on ion LET [71]. We thus set it constant, equal to $1.14\times 10^{-5}$ Gy^−1^ to yield an average number of 35 DSB/Gy/cell for low LET. This average number showed no dependence on nuclear geometry and little dependence on ion LET [77]. The number of DSBs in a monomer was rarely greater than 1, except for the case of high-LET radiation. 

### 4.4. DSB Repair

The next step consisted of modeling DSB repair over a 24 h time period. This corresponds roughly to the time left for a fibroblast to repair before inducing premature chromosome condensation [53,68], the time at which the fraction of remaining breaks starts reaching a plateau value [52]. Note that lymphocytes are usually given more time to repair, typically around 50 h [18,19,20,21]. Each DSB led to the formation of two free ends. The current RITCARD version assumes that the number of breaks, N(t), follows a bi-exponential decay as a function of time, t [52,53],

(4) $$N\left(t\right)=N_{1}\exp\left(-\frac{t}{\tau_{1}}\right)+N_{2}\exp\left(-\frac{t}{\tau_{2}}\right),$$

where $\tau_{1}$ and $\tau_{2}$ are time repair constants. The bi-exponential decay of breaks has been reported by many investigators and suggests that simple breaks are repaired rapidly ($\tau_{1}$ = 1.7 h) while more complex breaks take longer to repair ($\tau_{2}$ = 23.7 h). The time constants were set based on experimental repair times measured in primary normal human skin fibroblasts (HSF42) [78]. To simplify the scope of this work, we kept the repair constants fixed regardless of the cell type. $N_{1}$ and $N_{2}$ were not explicitly set, but breaks were categorized into simple and complex, based on a voxel energy threshold of 500 eV, allowing to reproduce experimental bi-exponential repair kinetics [52].

The repair algorithm then consisted of time steps, δt, (typically 1 s) over a period of 24 h. At each time step, a repair attempt was made for all free ends. Each pair of simple free ends was assumed to repair properly (one free end recombined with the free end originating from the same DSB) or to remain unrepaired, with a probability of proper repair equal to $\delta t/\tau_{1}$. Complex free ends had the additional outcome of improper repair. For one complex free end, the total probability of proper and improper repair was $0.5\cdot \delta t/\tau_{2}$, with the 0.5 factor accounting for the fact that each complex free end was counted twice in the complex repair algorithm. If the free end was repaired during a time step, then the Euclidian distance, r, between the selected free end and all other complex free ends was calculated. The probability of any two ends repairing was equal to,

(5) $$I=\frac{1}{W}\exp\left(-\frac{r^{2}}{\sigma^{2}}\right),$$

where W=50 and $\sigma^{2}=0.8$ μm^2^ are adjustable parameters that were empirically calibrated to match simple and complex exchange yields for gamma and 1000 MeV/n Fe irradiation [70]. The parameters can be adjusted to reflect change of kinetics of repair-deficient cells. Equation (5) reflects the fact that breaks further away from each other have a lower probability to recombine together. Using Equation (5), a list of probabilities for the given free end to react with any other free end is generated and used to sample one free end for the selected break to repair with, thus leading to either proper or improper repair.

### 4.5. Chromosome Aberrations

At the end of the 24 h period, the last step consisted of analyzing all the fragment sequences that were formed and classifying them in RITCARD. The classification includes intact chromosomes, properly repaired chromosomes, deletions, and CAs (translocation, inversions, dicentrics, rings, and simple or complex exchanges). The criteria used followed the same as those defined in [47,70] and are based on the work of [79]. Aberration types are not necessarily exclusive; for example, a ring can also be a dicentric. In this work, we focused on simple and complex exchanges. Simple exchanges (dicentrics and translocations) were defined as exchanges that involved 2 breaks, each coming from a different chromosome. A case is illustrated in Figure 15, where the 2 breaks involved in the simple exchange are circled. Complex exchanges were defined as exchanges that involved more than 2 breaks in 2 or more chromosomes.

For a given ion beam, we calculated CAs for 7 dose points ranging from 0.05 Gy to 1 Gy. Each dose point consisted of 10,000 histories in RITCARD. At the end of the simulation, for each dose point, we obtained an average number of exchanges and the statistical standard error. The dose response of simple or complex exchange frequency was then fitted by a linear quadratic (LQ) model,

(6) $$y\left(D_{av}\right)=\alpha D_{av}+\beta D_{av}^{2}.$$

$y\left(D_{av}\right)$ is the number of exchanges (simple or complex), and $D_{av}$ represents the average dose obtained by RITRACKS at the end of a simulation, which is close to the irradiation dose, D. The procedure used for the fit was described previously [73]. At the end of the procedure, we obtained a distribution of α and β values, out of which the average value and the standard deviation were computed. We also calculated the 95% confidence interval.

## 5. Conclusions

Chromosome aberrations are potential biomarkers that can be used to estimate space radiation cancer risks [11]. Over the past decades, the dependence of CA formation on heavy ion properties has been identified, with yields peaking for ion LET usually around ~100 keV/μm. Several studies [4,32,33,34,35,36] suggest that the nuclear geometrical properties, and more generally CA distribution within the nucleus, could also be a factor impacting experimental outcomes. This study, based on the Monte Carlo simulation models RITRACKS/RITCARD, investigates how the size and shape of the nucleus, and chromosome intermingling, affect simple and complex exchange yields, considering ion beams of LET ranging from 0.22 keV/μm to 195 keV/μm. In line with the conclusions of Ingram et al. [36], we observed that the nuclear geometrical properties and the 3D distribution of chromosomes are major parameters influencing CA predictions. Simulations obtained for spherical radii varying from 2 μm to 8 μm show that the volume of the nucleus has a major impact on both simple and complex yields. The beam orientation and shape also influence the yields, with flattened nuclei generally favoring the formation of simple over complex exchanges. Finally, we observed that chromosome intermingling overall increased the yield of both simple and complex exchanges. These findings can be attributed to the relative distribution of DNA DSBs within the nucleus that is heavily influenced by the chromosome distribution, and in turn affects CA yields as their formation depends on the distance between breaks. Our findings are consistent with some previously reported results on size effect (fibroblast vs. lymphocyte) [55,68] or shape and beam orientation effect [4,32,33,35]. Designing an experimental protocol that would allow the effect of the nuclear volume to be assessed while limiting the variation of other biological parameters would be interesting although not straightforward. While refinement is still required to match experimental results, our calculations suggest that the nuclear volume is a major parameter of CA predictions that could, at least in part, explain the apparent change in radiosensitivity between different cell lines.

These findings are important for several reasons. First, within the human body, cells can have varying sizes and shapes. If it was experimentally confirmed that geometrical properties (in particular nuclear size) are a major driver for CA formation, it would imply that different cell types could be more or less radio-sensitive depending on their size, and thus be more or less at risk. Second, as we show that the shape of the nucleus impacts CA formation, it is important to take such dependence into account for experiments, as several authors already reported [33,36]. In vitro protocols, such as those used for fibroblasts, usually measure CAs for a monolayer of adherent cells. Those cells have an elongated shape that can be different than for in vivo conditions. Additionally, they are usually irradiated along the shortest axis for which, in general, yields are different than for spherical nuclei. This shape effect should be accounted for to translate risks from in vitro studies to in vivo, which is important for space radiation risk assessment and might have implications for hadron therapy planning, as CAs are correlated with cell survival [80]. Many experimental studies investigating CAs report the nuclear irradiation area, but not the nuclear thickness (oriented along the beam axis). As the thickness can depend on the experimental protocols even for a given cell line, our findings show that it is important to fully characterize nuclear geometrical properties to allow inter-comparison and modelling.

## Figures and Tables

**Table 1:** List of nuclear geometries considered in this work. x, y and z represent the semi-axes of the nucleus (spherical or ellipsoidal). The beam is oriented along $R_{z}$. A represents the irradiated surface, and V is the volume of the nucleus. The sizes in bold represent experimental AG1522 [32] and 82-6 fibroblast sizes [53,54].

					Average Nuclear Ion Traversal/1 Gy		
x (µm)	y (µm)	z (µm)	A (µm^2^)	V (µm^3^)	H 1000 MeV/n	Si 170 MeV/n	Fe 450 MeV/n
**Volume study**							
2	2	2	12.6	33.5	356	0.8	0.4
3	3	3	28.3	113.1	802	1.8	0.9
4	4	4	50.3	268.1	1426	3.2	1.6
5	5	5	78.5	523.6	2228	5.0	2.5
6	6	6	113.1	904.8	3208	7.1	3.6
7	7	7	153.9	1436.8	4366	9.7	4.9
8	8	8	201.1	2144.7	5703	12.7	6.4
**Shape study**							
Fibroblast 82-6							
12.51	12.51	1.00	492.0	656	13,954	31.0	15.7
8.85	8.85	2.00	246.0	656	6978	15.5	7.9
7.23	7.23	3.00	164.0	656	4651	10.3	5.2
6.26	6.26	4.00	123.0	656	3489	7.8	3.9
5.60	5.60	5.00	98.4	656	2791	6.2	3.1
5.39	5.39	5.39	91.3	656	2589	5.8	2.9
Fibroblast AG1522							
8.81	8.81	0.50	243.6	162	6909	15.4	7.8
6.23	6.23	1.00	121.8	162	3454	7.7	3.9
4.40	4.40	2.00	60.9	162	1727	3.8	1.9
3.59	3.59	3.00	40.6	162	1151	2.6	1.3
3.38	3.38	3.38	36.0	162	1021	2.3	1.2
**Beam orientation study**							
Fibroblast 82-6							
**10.22**	**5.11**	**3.00**	**164.0**	**656**	4652	10.3	5.2
3.00	10.22	5.11	96.3	656	2731	6.1	3.1
5.11	3.00	10.22	48.2	656	1366	5.8	1.5
5.39	5.39	5.39	91.3	656	2589	3.0	2.9
Fibroblast AG1522							
**9.90**	**4.45**	**0.88**	**138.4**	**162**	3926	8.7	4.4
0.88	9.90	4.45	27.4	162	776	2.3	0.9
4.45	0.88	9.90	12.3	162	349	1.7	0.4
3.38	3.38	3.38	36.0	162	1021	0.8	1.2

**Table 2:** α (Gy^−1^) and β (Gy^−2^) coefficients for simple and complex exchanges for spherical and ellipsoidal AG1522 and 82-6 fibroblast nuclei irradiation by 170 MeV/n Si ion beam along the different semi-axes (R for the spherical shape and x>y>z for the ellipsoidal shape. w/o CD: without chromosome domain constraint; w CD: with chromosome domain constraint.

	Simple				Complex			
	$\alpha_{w/o\;CD}$	$\alpha_{w\;CD}$	$\beta_{w/o\;CD}$	$\beta_{w\;CD}$	$\alpha_{w/o\;CD}$	$\alpha_{w\;CD}$	$\beta_{w/o\;CD}$	$\beta_{w\;CD}$
AG1255								
$R_{\mathrm{AG}1522}$ = 3.38 μm	0.29	0.13	−0.09	−0.03	1.78	0.54	0.46	0.36
$x_{\mathrm{AG}1522}$ = 9.9 μm	0.17	0.04	−0.02	0.00	1.94	0.05	0.16	0.08
$y_{\mathrm{AG}1522}$ = 4.45 μm	0.20	0.08	−0.04	−0.01	1.70	0.27	0.28	0.11
$z_{\mathrm{AG}1522}$ = 0.88 μm	0.37	0.29	−0.08	−0.04	1.04	0.54	0.56	0.42
82-6								
*R*_82-6_ = 5.39 μm	0.44	0.35	−0.07	−0.04	0.44	0.05	0.22	0.11
*x*_82-6_ = 10.22 μm	0.42	0.27	−0.07	−0.05	0.39	0.00	0.18	0.07
*y*_82-6_ = 5.11 μm	0.45	0.32	−0.08	−0.03	0.42	0.05	0.17	0.08
*z*_82-6_ = 3.00 μm	0.44	0.39	−0.05	−0.04	0.33	0.16	0.14	0.11

**Table 3:** List of experimental nuclear sizes referenced in the literature. Experimental measurements are in bold with standard deviation values in parenthesis when available. Calculated values are in italic as an indication and assuming that the nucleus has a spherical/ellipsoidal shape.

Ref.	Cell Line	X Semi-Axis (µm)	Y Semi-Axis (µm)	Z Semi-Axis (µm)	Irradiation Area (µm^3^)	Volume (µm^3^)
**Normal human lymphocytes**						
[33] ^a^	Blood (1 donor)	**3.5–5**	**3.5–5**	**1.5**	*38–79*	*77–157*
		**2.75**	**2.75**	**2.75**	*24*	*87*
[26] ^b^	Blood (5 donors)	**2.05–3.55**	*13.2–39.6*	*11.5–59.7*		
**Normal human fibroblast**						
[54] ^c^	82-6	**8.0**	**5.0**		*126*	
[53]	82-6	*7.23*	*7.23*		**164 (10)**	
[31]	AG1522 (male foreskin)	**4.7 (<0.5)**		**0.55 (<0.05)**		
[55]	AG1521	*6.43*	*6.43*	**0.6**	**130**	*104*
[32]	AG1522	**9.9 (0.9)**	**4.45 (35)**	**0.88 (0.1)**	*138*	*162*
[56]	AG01522	*8.18*	*8.18*	**1.5**	**210 (20)**	
[57]	AG1522	*6.77*	*6.77*	**0.6**	**144 (45)**	*115*
[58]	AG1522	*7.36*	*7.36*		**170**	
[27]	Not Specified	**9.85**	**7.1**	**2.5**	*220*	*732*
[59]	HF19 (female fetal lung)	*6*	*6*	*6*	**113**	**904**
[60]	HF19	*8.41*	*8.41*		**222 (56)**	
[61]	HF19	*7.76*	*7.76*		**189**	
[62]	HF19			**<1.75**		
[63]	HFL-III (male fetal lung)	*6.74*	*6.74*		**142.9 (1.9)**	
[29]	HCA2 (male foreskin)	*7.6*	*7.6*	**1.25**	**180**	*302*
[64]	HF12 (male fetal lung)	*8.46*	*8.46*	**1.93 (0.28)**	**225 (106–444)**	*579*
[28]	HLF-CLL (male lung)	**16.1 (2.35)**	**9.0 (0.95)**		*455*	
[63]	NB1RGB (male skin)	*7.41*	*7.41*		**172.3 (2.8)**	
[25]	Skin biopsy (male)	**10**	**5**	**2.5**	*157*	*524*
[30]	Male foreskin (6 donors)	**8.36–9.15**	**6.42–7.83**	**2.02–3.17**		**492–1010**
**Normal human epithelial/endothelial cells**						
[27]	Endothelial	**9.55**	**5.5**	**1**	*165*	*220*
[4]	Bronchial Epithelial	**5.8**	**5.8**	**1.7**	**105**	*240*
**Hamster fibroblast**						
[65]	V79 (male)	*6.00*	*6.00*	**2**	**113 (34)**	*377*
[66]	V79 (late S)	*8.41*	*8.41*		**222**	
[66]	V79 (asynchronous)	*6.68*	*6.68*		**140**	
[66]	V79 (G1/S)	*7.25*	*7.25*		**165**	
[57]	V79	*4.95*	*4.95*	**1.9**	**77 (25)**	*195*
[67]	V79	*6.98*	*6.98*		**153**	
[60]	V79	*6.53*	*6.53*		**134 (36)**	

^a^ Dimensions provided by Schmid et al. [33] are for attached cells (flattened ellipsoid) or settled cells (sphere). ^b^ Calculations of area/volume were approximated with a spherical geometry, although authors reported an average major to minor axis ratio of 1.1–1.3. ^c^ Values extracted from microscopy images.

**Table 4:** Ion beams investigated in this study. The energy is provided in MeV/n, the LET in keV/μm, and R represents the projected range in water, in cm. Values were obtained with Stopping and Range of Ions in Matter (SRIM, http://www.srim.org/, accessed on 19 August 2021).

Ion	^1^H^1+^		^4^He^2+^			^12^C^6+^	^16^O^8+^			^28^Si^14+^			^48^Ti^22+^	^56^Fe^26+^		
**Energy**	1000	250	1000	250	150	290	325	250	128	600	300	170	600	1000	600	450
**LET**	0.22	0.39	0.89	1.56	2.2	12.9	21.5	25	39	50	68.9	99	125	149.2	174	195
**R**	322	37.5	323	37.6	15.6	16.4	14.6	9.4	3.0	22	7.3	2.8	15.6	27.4	13.1	8.4

## Data Availability

The simulation results can be obtained by request to the corresponding author. The software RITRACKS that has been used to perform these calculations is available at https://software.nasa.gov.
